# Addition of Selenium Nanoparticles to Electrospun Silk Scaffold Improves the Mammalian Cell Activity While Reducing Bacterial Growth

**DOI:** 10.3389/fphys.2016.00297

**Published:** 2016-07-14

**Authors:** Stanley Chung, Batur Ercan, Amit K. Roy, Thomas J. Webster

**Affiliations:** ^1^Department of Chemical Engineering, Northeastern UniversityBoston, MA, USA; ^2^Wenzhou Institute of Biomaterials and Engineering, Wenzhou Medical UniversityWenzhou, China; ^3^Center of Excellence for Advanced Materials Research, King Abdulaziz UniversityJeddah, Saudi Arabia; ^4^Department of Bioengineering, Northeastern UniversityBoston, MA, USA

**Keywords:** silk, electrospinning, antibacterial nanoparticles

## Abstract

Silk possesses many beneficial wound healing properties, and electrospun scaffolds are especially applicable for skin applications, due to their smaller interstices and higher surface areas. However, purified silk promotes microbial growth. Selenium nanoparticles have shown excellent antibacterial properties and are a novel antimicrobial chemistry. Here, electrospun silk scaffolds were doped with selenium nanoparticles to impart antibacterial properties to the silk scaffolds. Results showed significantly improved bacterial inhibition and mild improvement in human dermal fibroblast metabolic activity. These results suggest that the addition of selenium nanoparticles to electrospun silk is a promising approach to improve wound healing with reduced infection, without relying on antibiotics.

## Introduction

Researchers in the tissue engineering field work toward repairing and/or regenerating damaged tissues and organs through a combination of biomaterial scaffolds, cell signaling moieties, and cell (Langer and Vacanti, 1993). The ideal tissue engineering scaffold closely mimics the physical and chemical makeup of the organ to be replaced and should serve as an artificial extracellular matrix to support cell growth and differentiation. Electrospun scaffolds closely mimic the physical composition of native extracellular matrix (ECM) morphology.

Electrospinning works by applying a high voltage field to a solution of polymer dissolved in a conductive solvent (Sill and von Recum, 2008). The voltage induces electrostatic repulsion within the polymer solution and forms a cone like structure, the Taylor cone, held together by the force balance of the electrostatic repulsion and surface tension. Eventually, the electrostatic repulsion overcomes the surface tension forces holding the polymer solution together. Once this critical limit has been reached, a polymer jet is formed out of the edge of the Taylor cone toward a positively charged collector, and the solvent is evaporated in the flight path from the cone to the collector, leaving a polymer matrix with fibers relevant to physiological regime. The physical parameters of the scaffold may be adjusted based on polymer composition, solvent mixture, voltage, and many other parameters used to create the matrix. Researchers also have a high degree of control over the fiber orientation of electrospun scaffolds by adjusting the type of collector. Because of these processing advantages, electrospinning has been researched for a variety of tissue engineering applications such as cardio (Hajiali et al., 2011; Liu et al., 2011; Du et al., 2012), bone (Shin et al., 2010; Cai et al., 2012; Frohbergh et al., 2012; Liu et al., 2014), neural (Wang et al., 2011; Guan et al., 2013; Kador et al., 2013; Prabhakaran et al., 2013; Baiguera et al., 2014; Irani et al., 2014), skin (Dhandayuthapani et al., 2010; Jin et al., 2011; Kuppan et al., 2011; Rnjak-Kovacina et al., 2011), tendon/ligament (Howell et al., 2004; Sahoo et al., 2010a,b; James et al., 2011; Cardwell et al., 2012), and stem cell expansion/differentiation (Shin et al., 2010; Sahoo et al., 2010a; James et al., 2011; Jin et al., 2011; Wang et al., 2011; Cardwell et al., 2012; Irani et al., 2014).

Electrospun scaffolds promote many beneficial cellular responses for tissue engineering and are generally better for cell proliferation and differentiation than 2D substrates. In particular, silk electrospun scaffolds demonstrate good responses as tissue engineering scaffold for wound healing (Wharram et al., 2010; Gil et al., 2013; Lee et al., 2014). Silk promotes collagen synthesis, re-epithelialization, wound healing, atopic dermatitis alleviation, and scar reduction (Ricci et al., 2004; Fini et al., 2005; Roh et al., 2006; Okabayashi et al., 2009). However, pure silk shows negligible or even negative antibacterial properties (Kaur et al., 2014). Previously, groups have loaded electrospun silk scaffold with silver nanoparticles to impart anti-bacterial properties (Kang et al., 2007). However, silver is a commonly used antibiotic that has become resistant in certain strains of bacteria (Silver, 2003).

Selenium nanoparticles are a novel antibiotic chemistry to which there is no known bacterial resistance (Tran and Webster, 2011, 2013; Wang and Webster, 2012, 2013; Shakibaie et al., 2015). Selenium is a common trace element in the body and is important to healthy nutrition, especially in the formation of selenoproteins (Andrews et al., 2011; Santhosh Kumar and Priyadarsini, 2014). Selenium has been suggested to have anticancer effects as well (Clark et al., 1996). Here, we doped selenium nanoparticles to electrospun silk scaffold to impart antibacterial properties to silk (Rockwood et al., 2011). Human dermal fibroblasts were used to determine the *in vitro* changes in metabolic activity while *Staphylococcus aureus* were used to determine the effects of the bacterial inhibition.

## Materials and methods

### Materials

*Bombyx mori* silk cocoons were obtained from Mulberry Farms (Fallbrook, CA). Formic acid was purchased from Sigma-Aldrich (Saint Louis, MO). Selenium nanoparticles were synthesized as described below.

#### Extraction of silk fibroin from *Bombyx mori* silk cocoons

Silk fibroin was prepared from *Bombyx mori* cocoons according to previously established protocols with minor modifications (Rockwood et al., 2011). *B. mori* silk cocoons were cut into small pieces and boiled in 0.02 M Na_2_CO_3_ (Sigma-Aldrich) for 30 min to remove the glue-like sericin coating layer from the structural fibroin protein which was then rinsed 3x with distilled water (diH_2_O). The obtained silk fibroin fibers were dried overnight, dissolved in a LiBr (Sigma-Aldrich) solution (9.3 M) at 60°C for 4 h, and dialyzed through a cellulose membrane (ThermoFisher, Waltham, MA, 3500, MWCO) across distilled water for 4 days. The obtained silk solutions were centrifuged thrice at 4200 g and lyophilized for 4 days before resuspending in formic acid for a final concentration of 8% silk/formic acid.

#### Electrospinning of silk/formic acid solution

Eight percentage of silk solution was then electrospun at 18,000 volts, room temperature, 0% relative humidity, and 60 cm to collector. These conditions were optimized to produce fibers with dimensions that resemble those from the native extracellular matrix. Afterwards, 70% methanol (Sigma-Aldrich) was used to treat the electrospun silk to prevent hydrolysis of the membrane. Treated silk membranes were dried overnight in the fume hood.

#### Selenium nanoparticle synthesis

0.1 M sodium selenite [Alfa Aesar, Ward Hill, MA, Na_2_SeO_3_(H_2_O)_5_] and 0.1 M glutathione, GSH (Alfa Aesar), (C_10_H_17_N_3_O_6_S) were added onto the treated membranes before 0.2 M sodium hydroxide (NaOH) was added to precipate the sodium nanoparticles. Finally, double distilled deionized water was added thrice to quench the reaction and wash the membranes.

### Specimen characterization

Imaging of the specimens was conducted with a Hitachi S4800 Tokyo, Japan Scanning Electron Microscope (SEM, Hitachi S4800 SEM, Tokyo, Japan). A 4.5 nm layer of platinum was sputter coated (Cressington 208; Cressington Scientific Instruments, Watford, UK) onto the membranes to provide a conductive surface. SEM analysis was conducted with a 3 kV accelerating voltage. Characterization was completed using both secondary electrons and backscatter electrons, which impart a stronger signal to heavier elements, such as selenium.

### Cellular assays

#### Mammalian cell activity culture and characterization

Passages 3–12 human dermal fibroblast (HDF, Lonza, Basel, Switzerland) were cultured in Dulbecco's Modified Eagle Medium (DMEM, Sigma Aldrich) supplemented with 10% fetal bovine serum (Hyclone, Logan, UT) and 1% penicillin/streptomycin (P/S, Sigma Aldrich) in a 37°C, humidified, 5% CO_2_/95% air environment.

MTS assay (Promega, Fitchburg, WI) was used to determine the metabolic cell activity of the HDFs. Before cell seeding, the electrospun silk scaffolds were washed with 70% ethanol (Sigma-Aldrich) before rinsing with double distilled deionized water. HDFs were cultured to ~90% confluence, rinsed with Dulbecco's phosphate-buffered saline without calcium chloride and magnesium chloride (dPBS, Sigma Aldrich), and detached from the tissue culture plate by using 0.25% trypsin-EDTA (Sigma-Aldrich). Detached cells were then centrifuged at 2000 r.p.m. and resuspended at a density of 50,000 cells/ml before seeding onto the silk scaffolds in a 96 well-plate at 100 μl in each well (5000 cells/well). The HDFs incubated for 1, 2, and 4 days. Afterwards, the medium was removed from the sample and 100 μl solution of 1:5 MTS dye with DMEM medium (v/v) were added to each well. Samples were placed back into the incubator for 2.5 h. to allow the MTS to react with the metabolic products of the adherent cells before reading in a SpectraMax M3 microplate reader (Molecular Devices, Sunnyvale, CA) at an absorbance wavelength of 490 nm. The absorbance values of wells containing only DMEM medium without cells were subtracted from the absorbance values of the wells containing cells. The metabolic activity of each well was compared with the metabolic activity of known numbers of cells by a standard curve constructed at the beginning of each trial.

#### Bacterial cell activity measurement

*Staphylococcus aureus* (ATCC-12600) were inoculated in 3% tryptic soy broth (TSB, Sigma-Aldrich) overnight. After 24 h., the *Staphylococcus aureus* were diluted with TSB until absorbance value reached 0.52 at wavelength of 562 nm. This corresponded with a cell density of 10^9^ colony forming units (CFU)/ml. Afterwards, the *Staphylococcus aureus* were diluted 1000x in TSB before seeding onto the silk samples in a non-treated 96 well-plate in 100 μl of solution (10^5^ CFU/well). After 24 h., the BacTiter Glo assay (Promega), a luciferase based ATP assay was used to quantify the amount of ATP present on the electrospun silk samples. BacTiter Glo reagent was added at the same volume as the medium in each well, 100 μl, at room temperature. The samples were inoculated at room temperature for 5 min. while the BacTiter Glo reagents solubilized the bacterial membrane, after which, the luminescence was measured using the SpectraMax M3. A standard curve was constructed to equate the luminescence readings with known ATP amounts.

### Statistics

All experiments were conducted in triplicate and repeated at least three times each. Analysis of variance and student's *t*-test were used to determine whether the differences in cellular activity over the different time periods were significant.

## Results and discussion

**Figure 1:**
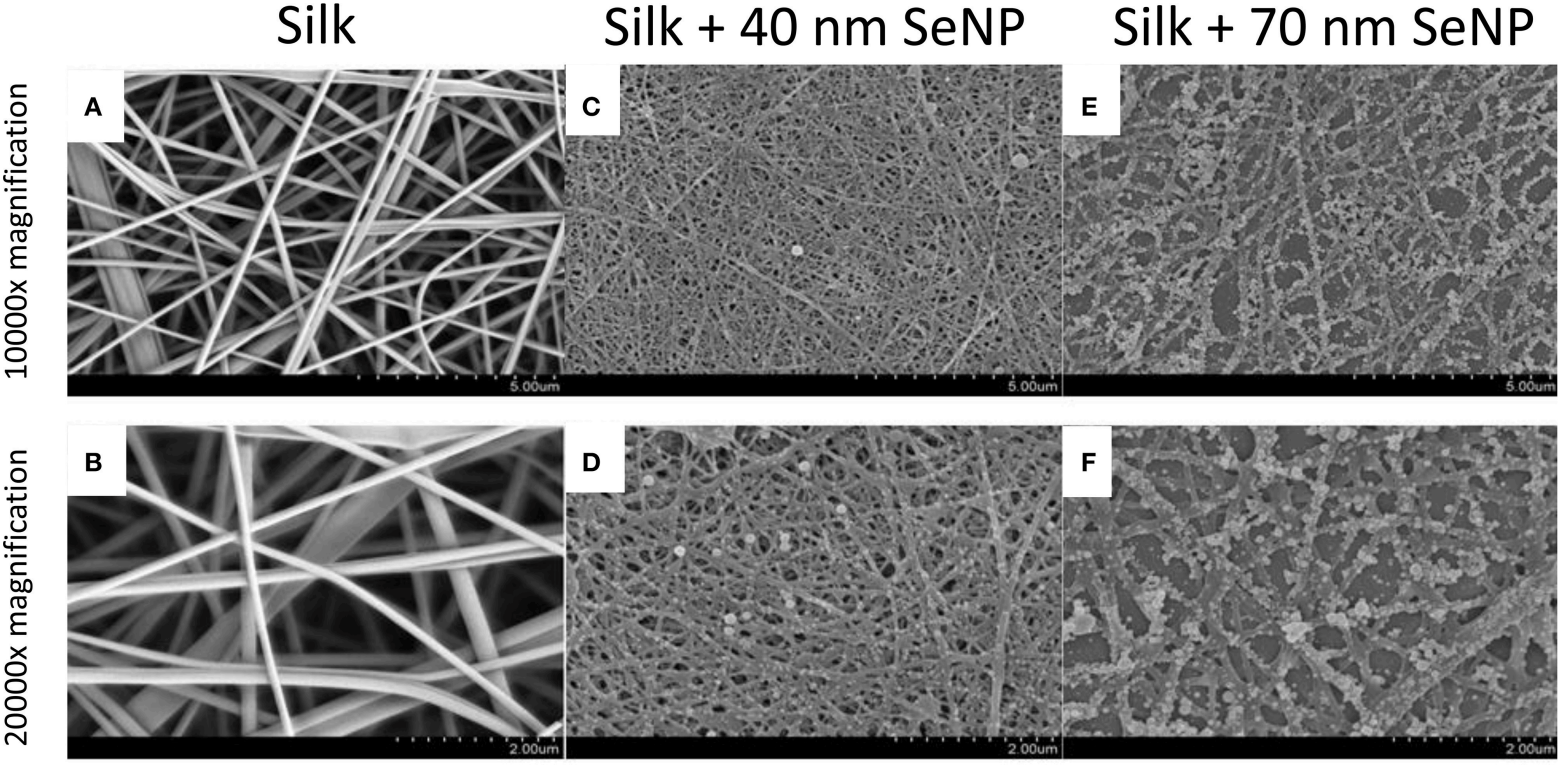
**Scanning electron microscopy (SEM) images of the electrospun silk scaffolds at 10,000x (A,C,E) and 20,000x (B,D,F) with 5 and 2 μm scale bars respectively**. The silk scaffolds without selenium nanoparticles are shown in panels **(A,B)**; with 40 nm selenium nanoparticle in panels **(C,D)**; and with 70 nm selenium nanoparticles in panels **(E,F)**.

To characterize the morphology of the electrospun silk scaffold, scanning electron microscope was used to visualize the surface of the nanocomposite. As shown in Figures 1A,B, the electrospun silk scaffolds contained fiber diameters ~100–200 nm and pore sizes ~2 μm. The silk scaffold contained unaligned fibers with very little beading and uniform thickness, demonstrating a morphology similar to those in the native extra-cellular matrix (ECM). The selenium nanoparticles (SeNP) were then reacted on the scaffold, causing a physisorption of the SeNP onto the silk scaffold. Two reaction conditions were chosen to deposit the SeNPs; SEM images showed that these produced two homogenous and distinct selenium nanoparticle populations: 40 (Figures 1C,D) and 70 nm (Figures 1E,F) nanoparticles.

**Figure 2:**
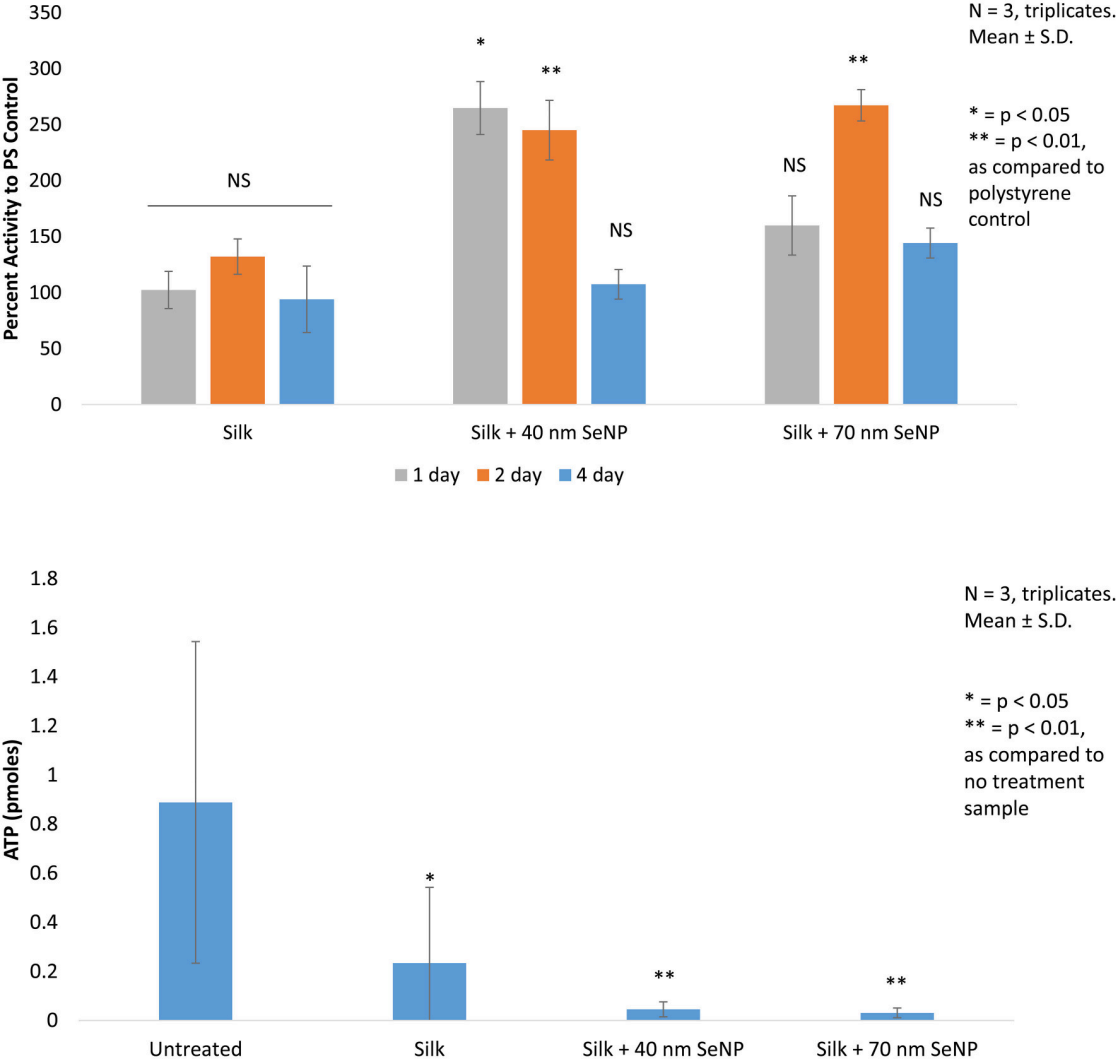
*****In vitro*** cellular activity are depicted. (Top)** The mitochondrial activity of human dermal fibroblast (HDF) grown on silk, silk +40 nm selenium nanoparticles, and silk +70 nm selenium nanoparticles are compared to activity of HDF grown on polystyrene tissue culture dish. All trials are conducted in triplicates, *N* = 3.^*^p < 0.05, ^**^p < 0.01 as compared to polystyrene control. **(Bottom)** The ATP activity of *Staphylococcus aureus* grown on the same sample groups as tested for the HDF is depicted. All trials are conducted in triplicates, *N* = 3.^*^p < 0.05, ^**^p < 0.01 as compared to no treatment sample.

First, *in vitro* viability tests were conducted using HDF cells. These cells were seeded onto the silk scaffold without selenium nanoparticles, the silk scaffolds containing the 40 and 70 nm selenium nanoparticles, and on regular polystyrene (PS) tissue culture plate to determine the change in growth of the HDF cells when grown on these substrates (Figure 2). Electrospun silk without addition of selenium nanoparticles produced statistically insignificant change (*p* > 0.05) in HDF activity as compared activity when grown on normal tissue culture plate across all 3 days tested. The silk/selenium nanocomposites produced significantly higher metabolic activity for HDF as compared to HDF grown on tissue culture plate. The addition of selenium nanoparticles significantly improved the metabolic activity of HDF, especially at the shorter term time points. At 1 and 2 days, silk scaffold doped with 40 nm selenium nanoparticles produced greater than two fold increase in metabolic signal, 264 and 245% on day 1 and 2, respectively, as compared to the tissue culture plate control while the 70 nm scaffold produced 160 and 267% on day 1 and 2, respectively. The day 1 (*p* < 0.05) and day 2 signal (*p* < 0.01) from the silk+40 nm SeNP scaffolds and the day 2 signal (*p* < 0.05) from the silk+70 nm SeNP scaffolds were statistically significant from the signal produced at the same time points on the tissue culture plates. Surprisingly, the silk scaffolds without nanoparticle addition did not produce a statistically significant improvement in HDF growth despite the ECM like morphology. There was mild improvement on day 2 (132% compared to control), but overall, the presence of silk alone did not significant improve HDF response. The short term improvement in activity may have plateaued by day 4 compared to the control, because the HDF may have reached confluency on the silk/selenium nanocomposites.

Finally, the bacteria results were accessed by ATP assay. Here, the performance of the silk scaffolds were compared to *Staphylococcus aureus* grown in solution in the polystyrene plate. Bacterial growth across all silk samples showed statistically significant reduction as compared to untreated samples grown in solution. Bacteria grown on silk without selenium nanoparticles showed a 74% reduction as compared with the control (*p* < 0.05). This was somewhat surprising, because this contradicted other reports found in literature (Kaur et al., 2014).

Addition of selenium nanoparticles significantly improved the bacterial load: the addition of the 40 nm selenium nanoparticles reduced bacterial load by 95% and the 70 nm selenium nanoparticles by 96% compared to the control (*p* < 0.01). The doping of selenium had achieved an additional reduction of 80% for the 40 nm selenium nanoparticle and 87% for the 70 nm selenium nanoparticle as compared to the silk samples. The 70 nm selenium nanoparticle produced an almost one log reduction in ATP content (0.88) as compared to the silk sample and an overall 1.4 log reduction compared to the control.

## Conclusion

This study showed for the first time the efficacy of doping selenium nanoparticles with silk to improve bacterial efficacy. Reaction conditions successfully synthesized two different sized populations of selenium nanoparticles onto electrospun silk scaffolds. These nanocomposites were then compared to silk scaffolds and normal tissue culture plates and were found to significantly improve both the mammalian cell response while reducing bacterial cell activity. Addition of the selenium nanoparticles significantly improved the short term human dermal fibroblast metabolic activity while reducing the ATP content of *Staphylococcus aureus*. Together, these results suggest that selenium nanoparticle may selectively enhance mammalian cells functions while killing or reducing the bacterial load. In summary, this study provides evidence of the potential value of the use of selenium nanoparticles in skin applications due to their selective activity. Future works will focus on determining the mechanism by which selenium nanoparticles achieve this selectivity and the scope of the selenium nanoparticles for inhibiting bacteria in skin applications.

## Author contributions

SC and BE made substantial contributions to conception and design, and acquisition of data, and nanalysis and interpretation of data. Equally contributed to planning and idea. AR participated in design of experiment/idea and revising the article. TW edited/revised it critically for important intellectual content and gave final approval of the version to be submitted and any revised version.

## Funding

The authors would like to thank Northeastern University for funding and facilities.

### Conflict of interest statement

The authors declare that the research was conducted in the absence of any commercial or financial relationships that could be construed as a potential conflict of interest.
